# Computing uveal melanoma basal diameters: a comparative analysis of several novel techniques with improved accuracy

**DOI:** 10.1186/s40942-018-0151-x

**Published:** 2019-01-09

**Authors:** Anthony B. Daniels, Kevin K. Veverka, Shriji N. Patel, LuAnne Sculley, Garvin Munn, Jose S. Pulido

**Affiliations:** 1 Department of Ophthalmology and Visual Sciences, Vanderbilt Eye Institute, Vanderbilt University Medical Center, 2311 Pierce Avenue, Nashville, TN 37232 USA; 2 Program in Cancer Biology, Vanderbilt University, Nashville, TN USA; 3 Department of Radiation Oncology, Vanderbilt University Medical Center, Nashville, TN USA; 4 Vanderbilt-Ingram Cancer Center, Vanderbilt University Medical Center, Nashville, TN USA; 5 Department of Ophthalmology, Mayo Clinic, 200 First Street, SW, Rochester, MN 55905 USA; 6 Department of Molecular Medicine, Mayo Clinic, 200 First Street, SW, Rochester, MN 55905 USA

**Keywords:** Uveal melanoma, Ocular tumors, Ultrasonography, Plaque brachytherapy

## Abstract

**Background:**

We sought to compare the accuracy of standard and novel echographic methods for computing intraocular tumor largest basal diameter (LBD).

**Design:**

Multicenter, retrospective cohort study.

**Subjects:**

All patients presenting with new diagnosis of uveal melanoma (UM).

**Methods:**

Ultrasounds were obtained for all patients, and axial length (AL) was measured for a subset of patients. LBD was calculated as: (1) a single chord measured on B scan ultrasound (one-chord method [1CM]), or (2) by subdividing the basal diameter into two chords, which were summated (two-chord method [2CM]), or (3) by a mathematically-derived formula (MF) based on geometric relationships. The accuracy of each method was then compared, and sensitivity of each technique to factors such as tumor size and AL were analyzed.

**Main outcome measures:**

Accuracy, robustness, correctness of predicted plaque size.

**Results:**

116 UMs were analyzed; 1CM-calculated LBD underestimated 2CM-calculated LBD by 7.5% and underestimated LBD by MF by 7.8%; 2CM and MF were tightly correlated (average LBD difference = 0.038%). At larger LBDs, 1CM underestimated 2CM and MF by a much greater percentage (*p* < 0.001). By linear regression, 1CM underestimated LBD compared to 2CM by 0.8% and underestimated LBD compared to MF by 1.2% for every 1-mm LBD increase (*p* < 0.001 for each). Increasing the number of ultrasound chords beyond two did not significantly impact LBD calculations. For eyes with AL within two standard deviations of the mean, AL did not impact plaque selection using MF. 1CM would have led to selection of an undersized plaque in 41% of cases compared to 2CM and would have misclassified half of all eyes that actually required enucleation. For tumors with LBD < 12 mm, 1CM does not significantly underestimate LBD.

**Conclusions:**

Tumor LBD by 1CM is an inaccurate means of determining actual LBD, especially for larger tumors. Using either 2CM or MF is much more accurate, especially for tumors > 12 mm, where a single chord on ultrasound is more likely to lead to incorrect, undersized plaque selection. Our MF can be applied with great accuracy even in cases where the AL of the eye is not measured, using the population average AL (23.7 mm), and the formula ${\text{LBD}} = 23.7\sin^{ - 1} ({{{\text{chord}}\;{\text{length}}} \mathord{\left/ {\vphantom {{{\text{chord}}\;{\text{length}}} {23.7}}} \right. \kern-0pt} {23.7}})$.

## Background

Since the multicenter Collaborative Ocular Melanoma Study (COMS) [1], radiotherapy has replaced enucleation for the treatment of the majority of medium-sized uveal melanomas (UMs). In fact, certain large melanomas [2, 3] or eyes with multiple melanomas [4] can also be salvaged with radiotherapy. However, successful local tumor control with brachytherapy depends on an accurate measure of the tumor size in order to ensure that a correct size plaque is selected that allows for adequate coverage at all tumor margins [5]. This is especially true in light of recent evidence that geographic miss, resulting in undertreatment of a tumor edge, is a primary cause of local treatment failure and tumor recurrence in UM [6–8].

**Fig. 1:**
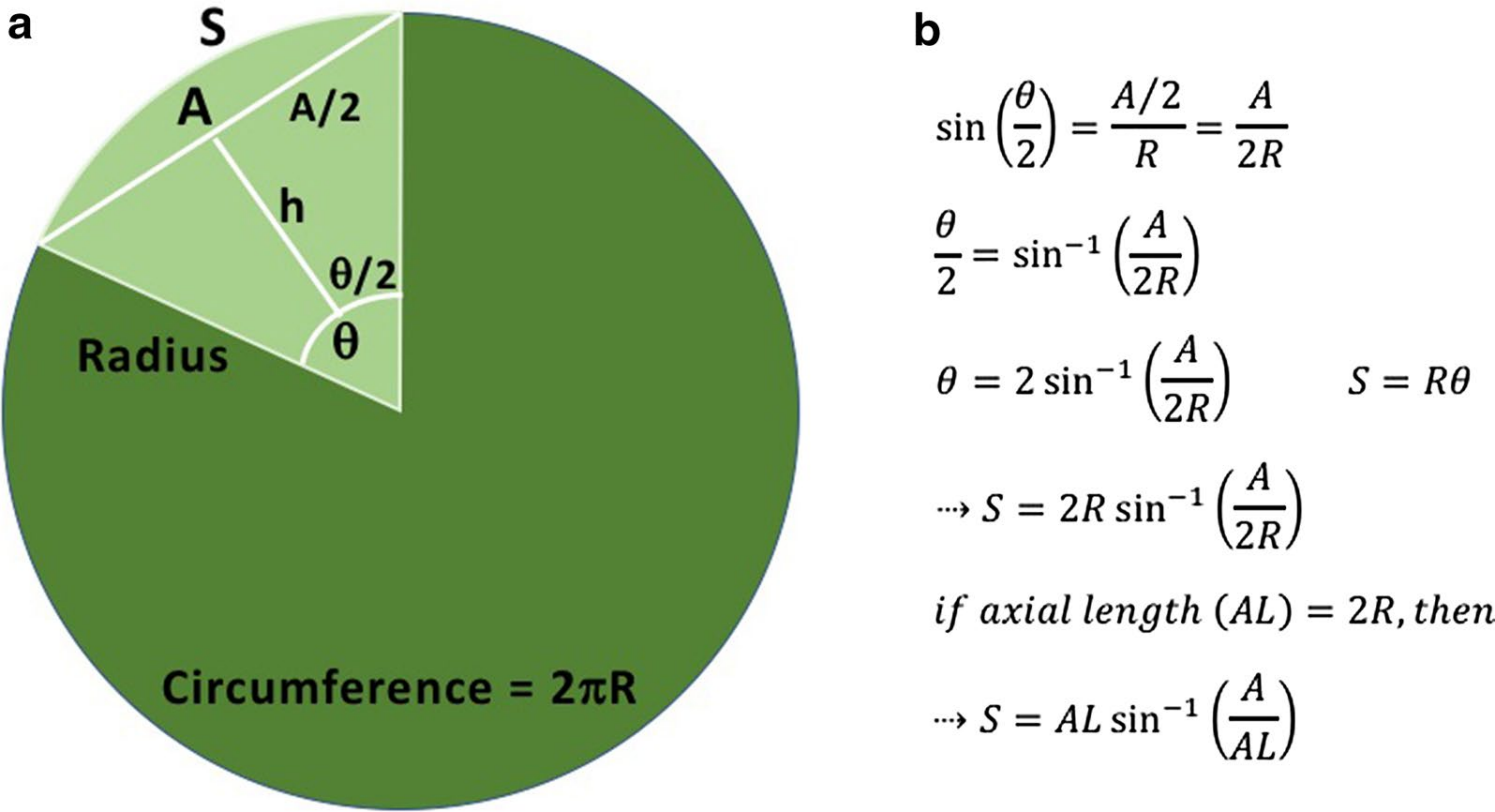
Geometric relationships between a chord length (*A*) and the arc (*S*) that it subtends; *R* represents the radius of the circle

There are several methods currently in use by clinicians to estimate tumor size [9–12]. Among these, studies have shown that ultrasonography with a single chord measured on the B scan is among the more commonly employed methods [9]. However, because of the roughly spherical shape of the globe, geometrically, this would be expected to underestimate the true basal diameter of the tumor, as measured at the sclera (Fig. 1).

We conducted an analysis to determine if, from a practical real-world perspective, a single-chord ultrasound measurement substantially impacts measured tumor largest basal diameter (LBD) and plaque selection. We also describe two simple alternative techniques (one empiric and one based on a mathematical geometric calculation) and demonstrate that these alternative methods better approximate true LBD. These novel methods of determining tumor LBD may assist surgeons in optimizing brachytherapy plaque selection and minimizing recurrences.

## Methods

### Patients

All patients with a new diagnosis of UM over the study period were included. At Mayo Clinic, the study period extended from February of 2013 to January of 2015; at Vanderbilt Eye Institute, the study period was from October of 2013 to February of 2015. Iris tumors were excluded from analysis, as were tumors that were so large as to fill the eye or preclude clear delineation of the tumor borders on ultrasonography.

The Institutional Review Boards at Mayo Clinic and at Vanderbilt University Medical Center each deemed that this study was considered “exempt.” This study was conducted in accordance with the Declaration of Helsinki and with the Health Insurance Portability and Accountability Act (HIPAA).

### Tumor measurements

**Fig. 2:**
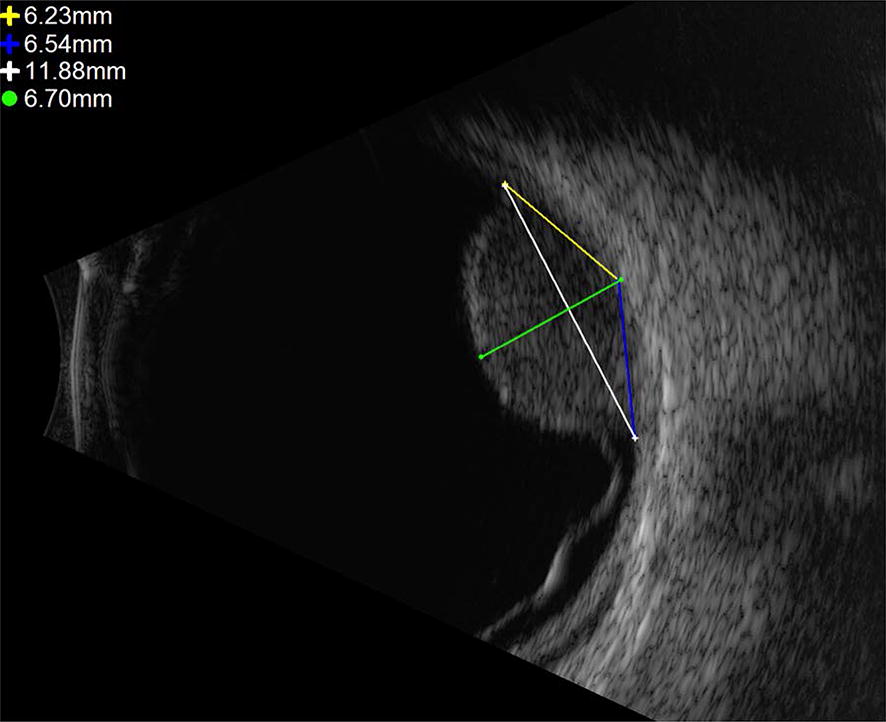
Relationship between one-chord method and two-chord method on B scan ultrasound

Ultrasounds were performed on the Ellex ultrasound machine or the Quantel Aviso in the B scan mode and were performed as part of standard-of-care evaluation for choroidal tumors. The LBD was determined in each axis (longitudinal and transverse) using the built-in caliper software. Tumor edge locations were confirmed by one of the two authors (ABD or JSP), and a single straight-line chord distance was measured between them (one-chord method [1CM]; Fig. 2). Subsequently, a two-chord method [2CM] was calculated by using the same anchoring endpoints, and the distance from one end to the midpoint (as measured at the base of the tumor) and from the midpoint to the second marked tumor edge. The LBD by 2CM was determined as the sum of these two shorter chords, each from a tumor edge to the midpoint.

For ciliary body tumors or ciliochoroidal tumors, ultrasound biomicroscopy (UBM) was also performed on the Ellex or Quantel machines. One-chord and two-chord measurements of the LBD were derived from the UBM in a similar fashion as for the traditional B scan mode that was used for posterior tumors.

Tumor height was measured on either the B scan (posterior tumors) or the UBM (for ciliary body tumors) using the caliper mode (Fig. 2). The greatest height was recorded as measured from the highest apex of the tumor to the choroid perpendicular to the base. Standardized A scan was used to confirm apical height. The sclera was not included in the measurement, consistent with standard protocols [10, 13].

### Globe measurements

Axial length (AL) was measured for a subset of patients in one of two ways. For patients who would be undergoing globe-conserving radiotherapy, the AL of both eyes was measured in the clinic by IOLMaster. For eyes in which the tumor did not involve the fovea, the AL for the affected eye was recorded. For eyes in which a macular tumor might artificially reduce the apparent AL, the AL for the contralateral eye was used, given the high correlation between the ALs of the two eyes of an individual [14].

For eyes undergoing primary therapeutic enucleation, the anteroposterior AL was measured directly on the freshly enucleated globe in the operating room. One millimeter was then subtracted from the measured length to make these measurements more consistent with the IOLMaster measurements in the larger radiotherapy group, since IOLMaster measures up to the inner surface of the retina at the fovea and not to the back of the sclera at this location. One millimeter was used as a standard scleral thickness across eyes, consistent with the protocol in the COMS [10].

### Mathematical derivation of tumor basal diameter

We approximated the eye to a sphere and, thus, the AL is equal to two times the radius of this sphere. Using the relationships marked in Fig. 1, the basal diameter, as measured along the arc of the base of the tumor, was derived as follows:

$$ \begin{aligned} & \text{Sin} \left( {\frac{\theta }{2}} \right) = \frac{{{\raise0.7ex\hbox{$A$} \!\mathord{\left/ {\vphantom {A 2}}\right.\kern-0pt} \!\lower0.7ex\hbox{$2$}}}}{R} = \frac{A}{2R} \\ & \frac{\theta }{2} = \sin^{ - 1} \left( {\frac{A}{2R}} \right) \\ & \theta = 2\sin^{ - 1} \left( {\frac{A}{2R}} \right)\quad S = R\theta \\ & \to S = 2R\sin^{ - 1} \left( {\frac{A}{2R}} \right) \\ & {\text{if}}\;{\text{axial}}\;{\text{length}}\;({\text{AL}}) = 2R,\;{\text{then}} \\ & \to S = {\text{AL}}\sin^{ - 1} \left( {\frac{A}{\text{AL}}} \right) \\ \end{aligned} $$

(Note that this calculation must be performed in Radians. All variables used in the above derivation refer to those defined in Fig. 1).

### Statistical analysis

All statistical analyses were performed using the STATA software package and Microsoft Excel.

## Results

### Patient and tumor characteristics

There were 116 UMs in 115 eyes of 115 patients evaluated and measured at the two study sites over the study period. One patient presented with two biopsy-confirmed choroidal melanomas in the same eye, as published previously [4]. All other patients had a single UM in a single eye; eight iris melanomas were excluded as was one patient whose eye was full of tumor, which precluded tumor measurements. In total, 107 posterior melanomas were included in this study.

There were 48 male and 58 female patients (*p* = 0.30). Average patient age was 64 years (range, 9-95). Fifty-six were right eyes and 50 were left eyes (*p* = 0.54). Eighty-six tumors were choroidal, and 10 (11.6%) of these choroidal tumors were juxtapapillary in location (posterior edge within 1 mm of the optic nerve head). Nineteen (17.8%) were ciliochoroidal and two (1.9%) involved the ciliary body only.

Of these 107 melanomas, 66 were treated by plaque brachytherapy, 23 were treated by enucleation, 12 were treated with stereotactic radiosurgery, and 4 were treated with transpupillary thermotherapy. One patient passed away prior to any treatment and one patient refused any treatment. ALs were available for 38 of these eyes. None of the eyes treated by plaque brachytherapy were found, at the time of plaque placement, to have an undersized plaque. None of the eyes treated by enucleation were found, upon evaluation of histopathologic sections, to have had a tumor of a size that could have fit a 22-mm COMS plaque (the largest COMS plaque available).

### Comparisons of basal diameter by various methods

Tumors had an average height of 4.8 mm (standard deviation [SD] 3.2, range 1.2–15.7 mm). By 1CM, the average LBD was 13.0 mm (SD 4.0), with a range from 4.6 to 21.0 mm. By 2CM, the average LBD was 14.2 mm (SD 5.0), with a range from 4.9 to 29.4 mm. For the subset of eyes with measured ALs (*n* = 38), the average LBD was 14.8 mm (SD 4.4), with a range from 5.7 to 22.7 mm as calculated by mathematical formula (MF), and the average LBD by 1CM for this same subset of tumors was 13.4 mm (SD 3.4) with a range from 5.6 to 19.6 mm. Regardless of method used, there was no statistical difference between measurements made in the longitudinal or the transverse direction (one chord, *p* = 0.30; two chord, *p* = 0.28).

**Fig. 3:**
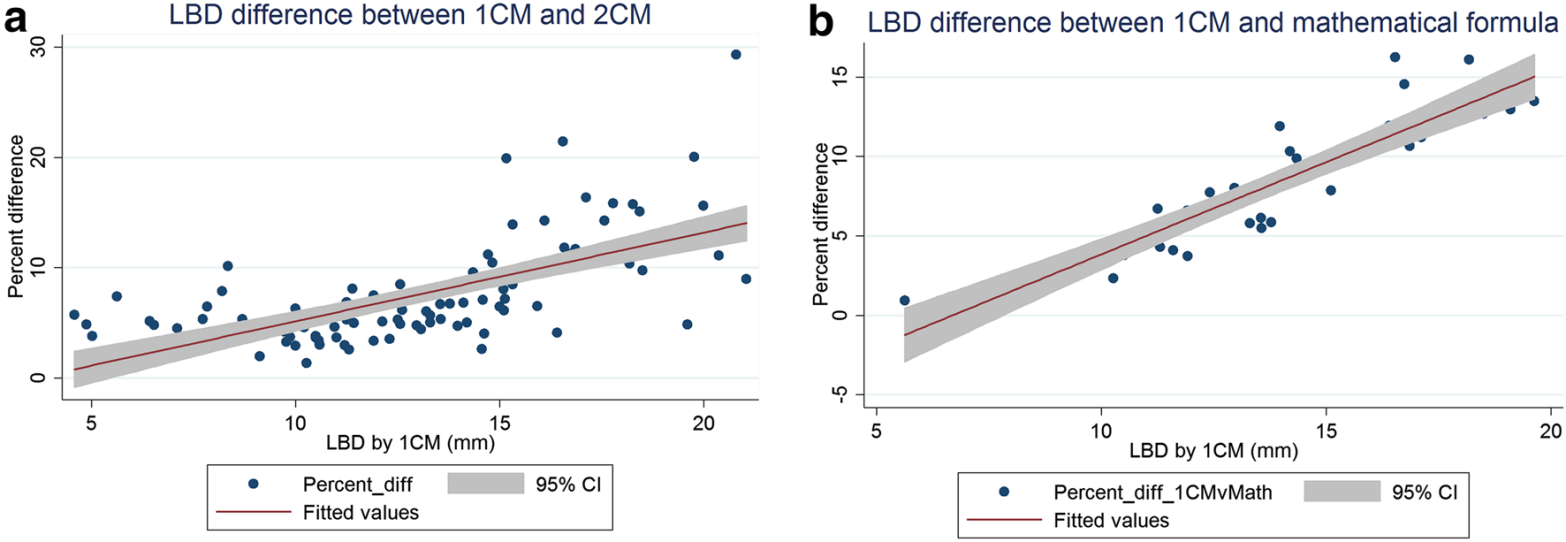
Difference in largest basal diameter measured by the one-chord method (1CM) and **a** the two-chord method (2CM), or **b** mathematical formula (MF), as a function of tumor size; one-chord method underestimated both other methods by an increasingly greater percentage for larger tumors. The grey area represents the 95% confidence interval

The LBD calculated by 1CM was always less than that calculated by 2CM or by the MF. On average, 1CM underestimated the LBD by 7.5% (range 1–29%), as compared to 2CM. On average, 1CM underestimated the LBD by 7.8% (range 1–16%), as compared to the MF-derived LBD. The 2CM method and the MF were much more tightly correlated, with an average LBD difference of only 0.038%, with neither one systematically greater or smaller than the other. At larger LBDs, 1CM underestimated 2CM and MF by a much greater percentage. By linear regression, 1CM underestimated LBD compared to 2CM by 0.8% for every 1 mm LBD increase (*p* < 0.001), and underestimated LBD compared to MF by 1.2% for every 1 mm LBD increase (*p* < 0.001; see Fig. 3). This makes sense mathematically, because at larger 1CM chord lengths, a single chord represents an increasingly worse approximation of actual arc length (representing actual tumor basal diameter, refer to Figs. 1, 2). Likewise, we determined the percent discordance between the 1CM and the 2CM and MF methods as a function of tumor height. We found that tumor height did not impact the degree of discordance between the LBDs calculated by the various methods.

### Determination of clinical implications for plaque selection

We wished to determine the real-world clinical implications of these various methods for computing LBD. We determined the correct plaque selection for the LBD of each tumor as determined by each method. We assumed COMS style plaques coming in 2-mm increment sizes in even number increments (i.e., 12 mm, 14 mm, 16 mm, etc.). First, we strictly applied the rule that each plaque must have 2-mm borders on each side, consistent with COMS study protocols and with American Brachytherapy Society guidelines [5, 10]. Thus, both a tumor with a 14.5-mm LBD and a tumor with a 15.5-mm LBD would be assigned a 20-mm plaque, but a tumor with a 13.5-mm LBD would be assigned an 18-mm plaque. Under these criteria, the 1CM method undersized the plaque compared to 2CM method in 41% of cases, resulting in a smaller plaque choice in each case. Both the number of misclassifications as well as the degree of misclassification (> 1 plaque size difference) increased for tumors with larger LBD. In 25% of cases, 2CM identified tumors too large for standard COMS plaques (i.e., would have required a plaque larger than the largest routinely available plaque [22 mm]). Of these tumors too large to plaque, 1CM failed to identify 50% of these large tumors; in these cases, using the 1CM would have resulted in the decision to proceed with plaque brachytherapy with a plaque that would have turned out to be too small for adequate coverage according to the American Brachytherapy Society or COMS guidelines.

### Sensitivity analyses

We performed several additional sensitivity analyses. First, we wished to determine if loosening the strict plaque selection criteria described above still leads to discordance in plaque size selection between the various methods. Specifically, does allowing a margin of only 1.5 mm per side make the plaque size selected more similar between the various methods? This was done to better approximate real life and to avoid the requirement to assign a 12.1-mm LBD tumor an 18-mm plaque. To do this, we assigned recommended plaque sizes based on 1CM assuming a 2-mm margin on each side and then assigned a plaque size based on 2CM, assuming only a 1.5-mm margin on each side. This avoided situations that could theoretically arise in which a tumor measuring 11.9 by 1CM and 12.1 mm by 2CM would be assigned discordant plaques of 16 mm and 18 mm, respectively. Even using these less-restrictive criteria and only requiring 1.5-mm margins around the tumor, 1CM still undersized 17.3% of plaques compared to 2CM.

We next assessed the effect that AL had on the MF. As expected based on the nature of the formula, shorter eyes led to greater-calculated basal diameters; however, this difference was minimal for all but the largest tumors. As demonstrated in Table 1, even for eyes with ALs two SDs above or below the mean [14], the range of calculated tumor LBDs is very narrow for all tumors that could potentially be treated with routinely available plaques. The range only begins to extend for very large tumors (where even a single-chord measurement is 18 mm or greater); and in this circumstance, regardless of the length of the eye, plaquing would not be indicated. Therefore, there is no combination of AL or tumor size where the surgeon would be led to select a different size plaque depending on the AL.

**Table 1:** Relative insensitivity of our mathematical formula to variations in axial length of the eye

Axial length	Largest basal diameter (by length of single chord measurement)						
	6 mm	8 mm	10 mm	12 mm	14 mm	16 mm	18 mm
21.9 mm (− 2 SD)	6.08	8.19	10.38	12.70	15.19	17.94	21.13
22.8 mm (− 1 SD)	6.07	8.17	10.35	12.64	15.07	17.74	20.75
23.7 mm (average)	6.07	8.16	10.32	12.58	14.98	17.56	20.44
24.6 mm (+ 1 SD)	6.06	8.15	10.30	12.54	14.89	17.42	20.19
25.5 mm (+ 2 SD)	6.06	8.14	10.28	12.49	14.82	17.30	19.98
Maximum difference (− 2 SD to + 2 SD)	0.02	0.05	0.10	0.21	0.37	0.64	1.15
Plaque selection	All same	All same	All same	All same	All same	All same	All same (enucleation)

The implication is that the actual axial length need not be measured, and the population average axial length (23.7 mm) can be substituted

mm, millimeters; SD, standard deviation

The implication of the above finding is that, except perhaps in cases of extreme pathologic myopia (> 2 SD above the mean) or nanophthalmos (> 2 SD below the mean), our MF can be used assuming an average AL (23.67 mm) [14] for the eye; therefore, a true clinical measurement of AL is not necessary. Thus, our formula can be reduced to the simpler form below:

$$ {\text{Calculated}}\;{\text{LBD}} = 23.67\sin^{ - 1} \left( {\frac{{{\text{Single}}\;{\text{chord}}\;{\text{length}}}}{23.67}} \right) $$

(Note that this calculation must be performed in Radians).

To confirm that this is truly the case, we validated this by recalculating the mathematically-derived tumor LBD for the 38 tumors with known ALs but, this time, assuming that the AL for each eye was the population average (23.67 mm) [14]. Replacing the actual measured AL with the population average AL resulted in an average difference in the mathematically-calculated tumor LBD of only − 0.21 ± 0.40 mm.

We next tested this hypothesis (that the MF can be used assuming an average AL) by validating this using data from 68 posterior melanoma-bearing eyes for which no AL data was available. This number was then compared to the 2CM measurements obtained for these same tumors/eyes. In this second validation cohort of melanoma-bearing eyes in which no ALs were known, we found that using the population average AL in our MF generated LBD measurements only 1.15 ± 4.00% different from that measured by 2CM.

Next, we wished to determine if increasing the number of ultrasound chords beyond two provided any meaningful advantage. The largest commonly-available COMS plaque is 22 mm, fitting a tumor with LBD of 18 mm, using the strict criteria. Using two chords, each chord could have a maximum length of approximately 9 mm. We, therefore, studied the subset of 16 tumors with LBD < 9 mm by 1CM. Among these small basal diameter tumors, the difference between 1CM and 2CM was always < 1 mm and always < 10% (average 0.38 mm, SD 0.21 mm). Similarly, by MF, for any eyes with ALs in the normal range (for the 2nd through 98th percentiles, or two SDs from the mean), a tumor with LBD by 1CM measured at 9 mm would be expected to have an LBD of 9.23 mm in an eye of average length, with a range between 9.27 mm for eyes with ALs two SDs below the mean and 9.20 mm for eyes with ALs two SDs above the mean. Thus, even among the largest tumors, subdividing additional chords beyond two does not increase sensitivity or accuracy by an appreciable amount.

## Discussion

We conducted a retrospective multi-institution analysis of patients with UM and compared methods for determination of the LBD: (1) by a single ultrasound chord measured (1CM) between the two ends of the tumor, (2) by dividing the base of the tumor on ultrasound and measuring multiple chords along the arc of the base of the tumor between the two ends of the tumor (2CM), and (3) by a mathematically-derived formula (MF) to calculate the theoretical basal diameter along the sclera based on the eye’s AL and the single-chord measurement. We found that the commonly-used single-chord ultrasound measurement of LBD underestimated true LBD, which was represented by an arc along the scleral edge of the tumor. Specifically, 1CM underestimated both 2CM and MF in all cases, and this difference became all the more clinically significant for larger-diameter tumors. In contrast, 2CM and MF remained extremely close to each other, even for very large tumors. While 1CM performed increasingly poorly as tumor diameter increased, neither tumor height nor AL affected the performance of each method.

There were certain assumptions underlying our analyses. There was no gold standard for tumor basal diameter measurement. While we have measured the diameter of the tumor shadow intraoperatively using a length of suture, this is imprecise, impractical, and cannot be performed ahead of time to assist with plaque size selection. Therefore, we started out with the assumption that 2CM and MF were more likely to represent the true basal diameter and that, to the degree that 1CM deviated from these, 1CM was more likely to be incorrect. There were several logical reasons why this assumption makes sense. First, there is no risk that 2CM will overestimate the arc that represents the true LBD of the tumor. Geometric relationships dictate that the chord or chords that approximate an arc along the surface of a circle will always have a sum less than that of the arc itself (Fig. 1). In fact, the sum of an infinite number of infinitely small chords would equal the length of the arc they subtend. Thus, we could be sure that the multi-chord method did not *over*estimate the length of the arc. We did not have such a priori assurances regarding the LBDs calculated by the MF. However, we found that 2CM and MF were extremely close to one another in all cases and, therefore, the concern that MF would overestimate LBD turned out not to be an issue.

The question still arises as to whether 2CM and MF are actually oversizing the plaques, and 1CM is actually the correct method. Apart from the theoretical considerations discussed above and demonstrated geometrically in Fig. 1, we tested this alternative hypothesis. We wished to determine if, rather than 1CM failing to identify eyes that should have undergone enucleation rather than plaque placement, 2CM and MF actually overcalled the need for enucleation. To do this, we determined the actual LBD on enucleation specimens based on the histopathologic sections, as was performed in the COMS study [9]. In no case was the actual histopathologic LBD small enough that the largest-available COMS plaque (22 mm) would have adequately covered the tumor. Thus, 2CM and the MF are *not* unnecessarily indicating enucleation for some tumors.

For tumors with measured single-chord LBDs below 14 mm, there was never a discordance of > 10% between either the 1CM and the 2CM methods or the 1CM and the MF methods. However, this still theoretically represents a difference of up to 1.4 mm (10% of 14 mm), which would almost certainly affect plaque selection (see “Determination of clinical implications for plaque selection” section). Below 12 mm, there was never a difference of > 1 mm between the LBD as determined by 1CM or 2CM measurements or between the LBD determined by 1CM and the MF method. Thus, for tumors with a single-chord length basal diameter on B scan ultrasound of < 12 mm, it is not necessary to correct with either the 2CM or the MF.

Another limitation of our MF model is that we approximated the eyeball to a sphere. This was a fair approximation, as the population average AL and transverse diameter of the human eye are quite similar. From a practical point of view, in clinic it is much easier to determine the AL of an eye than to try to measure the transverse diameter of an eye in situ. However, to more rigorously challenge the assumption that the globe can be approximated to a sphere using the AL as the sphere’s diameter, we recalculated the LBD using the MF assuming a two-compartment model. Specifically, we assumed the anterior chamber was a second hemisphere in front of the main sphere of the posterior segment. We selected this type of two-compartment modeling since it is the basis for many of the commercially available radiation planning software packages in use for calculating UM brachytherapy dosimetry. We assumed an average (pseudophakic) anterior chamber depth of 4 mm. Thus, the AL imported into all sections of the MF was reduced by 4 mm, as seen in the below equation:

$$ S = \left( {{\text{AL}} - 4} \right)\sin^{ - 1} \left( {\frac{A}{{{\text{AL}} - 4}}} \right) $$

In our cohort, utilizing this more complex two-compartment model, which accounts for the true non-spherical nature of the globe, results in a difference between the two-compartment model and the one-compartment MF of only 2.91 ± 3.99%. As expected, this discrepancy was slightly more evident in longer eyes. Thus, there is no clinically-meaningful benefit to utilizing a more complex two-compartment model, and the eye-as-a-sphere approximation is mathematically valid.

**Fig. 4:**
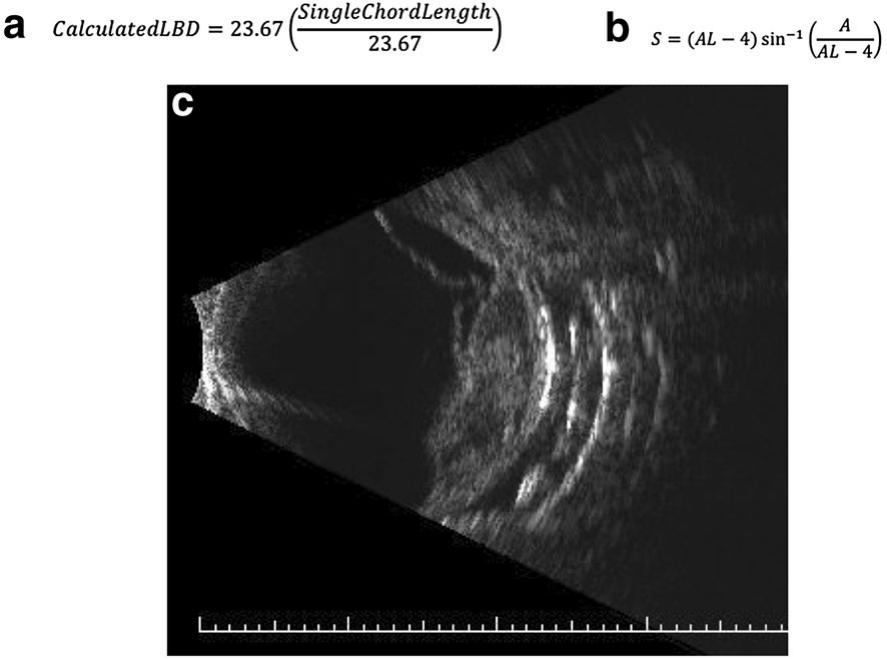
Intraoperative ultrasound of an undersized plaque, demonstrating inadequate margins at the tumor borders (photograph courtesy of referring surgeon). The tumor recurred and was subsequently referred to one of the authors (ABD) for treatment

The importance of the correct determination of tumor LBD cannot be overstated. There is evidence that geographic miss, where a tumor edge remains incompletely covered either due to misalignment or inadequate plaque size, is the leading cause of tumor recurrence [6–8]. Intraoperative ultrasonography has been shown to improve plaque alignment and, therefore, to increase successful tumor treatment [6, 15–17]; however, this does not help in cases where the selected plaque is too small for a given tumor, even if centered perfectly (Fig. 4). It is known that recurrent UMs are more aggressive and more likely to metastasize [18, 19]; therefore, it is crucial to minimize recurrences by ensuring adequate tumor coverage on all sides.

Similarly, the number of plaque reclassifications is not insignificant. We found a discrepancy between 1CM and the other two methods in > 40% of cases, meaning that > 40% of tumors would not have the full recommended 2-mm plaque margins. Perhaps more importantly, the commonly used 1CM failed to identify half of all the eyes for which enucleation was indicated based on the various other methods. This is critical information given that previous studies have shown that most surgeons use ultrasound as a determining factor in tumor sizing and plaque selection, and a large proportion of surgeons use it as the *primary* measure of tumor size [9].

**Fig. 5:**
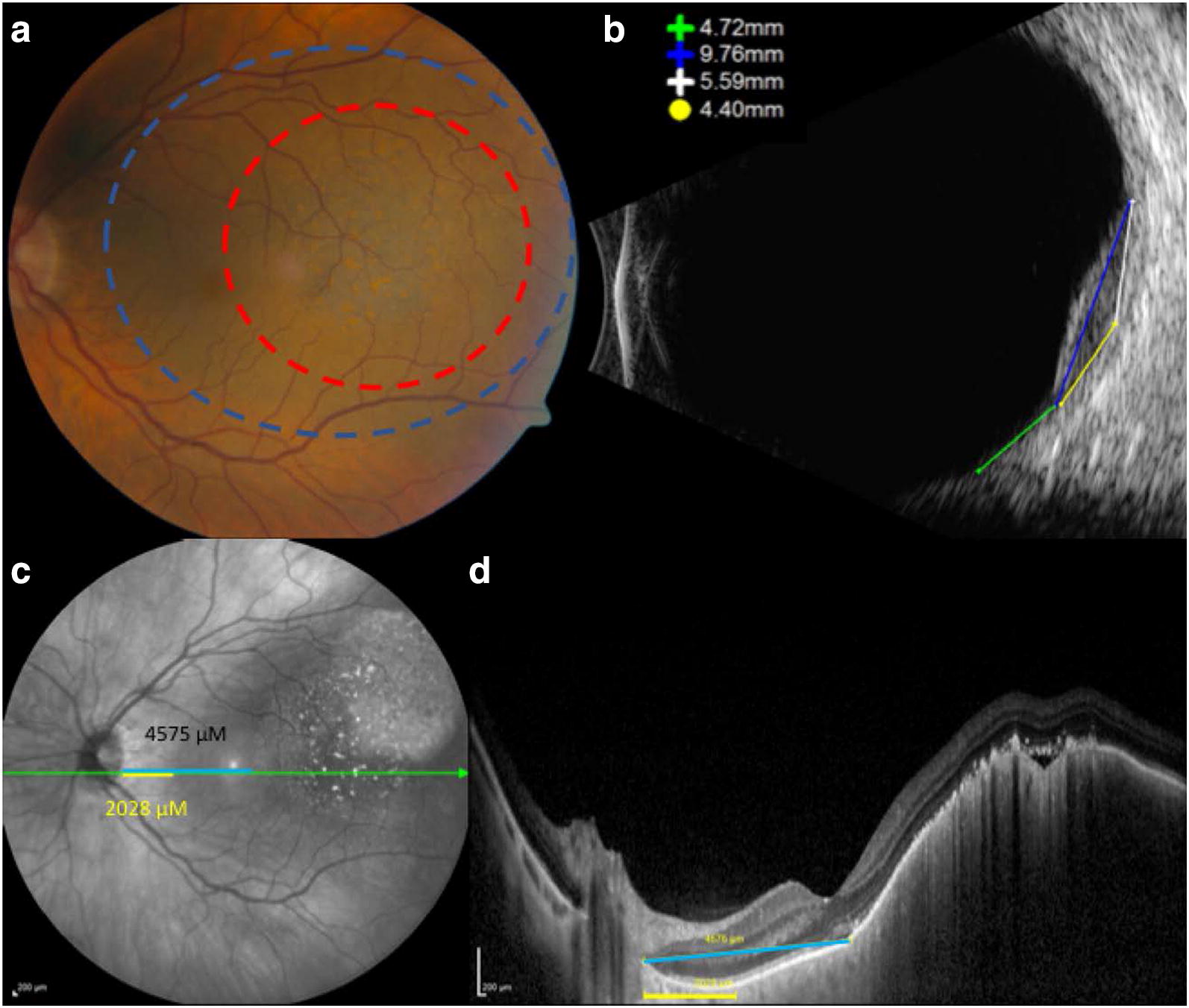
Flat pigmented component of a tumor surrounding the ultrasonographically-measurable component. **a** Fundus photograph of tumor demonstrating area of flat pigment (blue dashed line) surrounding the elevated portion (red dashed line) of the tumor. **b** B scan ultrasound of the same tumor shown in part **a**, demonstrating that the area of choroidal tumor elevation measurable on B scan is approximately 4.7 mm away from the optic nerve (optic nerve shadow is seen at the bottom of the panel). **c**, **d** OCT imaging demonstrating that the distance from the optic nerve to the edge of the pigment is much less than to the edge of the elevated tumor. The OCT cut in part **d** is shown as a green line in the infrared image seen in part **c**. The yellow and blue lines in **c** and **d** represent the distance from the edge of the optic nerve head to the edge of the flat pigmented portion of the tumor (yellow lines) and to the edge of the elevated portion of the tumor (blue line). The distances shown in micrometers were calculated using the inbuilt Spectralis caliper measuring tool. Note how close the B scan measure of the distance from the elevated portion of the tumor (4.72 mm to the optic nerve head) is to the OCT measure of the distance from the elevated portion of the tumor (4.58 mm to the optic nerve head). This figure illustrates that the flat pigmented portions of the tumor may not be visible on ultrasound, and so they need to be added and incorporated into the overall determination of plaque size in clinical practice

It is important to note that our 2CM and MF methods only improve the accuracy of the ultrasonographically-visible portion of the tumor. Certain tumors may have ophthalmoscopically evident (but ultrasonographically-invisible) areas of flat pigment at the edges of a tumor (Fig. 5). Any time that determination of basal diameter includes an ultrasound measurement component, it is important to assess these flat pigmented areas ophthalmoscopically, as well as the contour of the tumor on the ultrasound B scan, to determine if they are included in the area measured with the calipers on ultrasound. In these cases, enhanced depth imaging optical coherence tomography can sometimes be useful to identify elevation that is beyond the limits of detection of ultrasound (Fig. 5) [20]. For truly flat pigmented portions of the tumor, these areas would not be measurable on the ultrasound. In such cases, the size of the adjacent flat pigmented area of the tumor must be approximated based on fundoscopy and added to the ultrasound measurement of basal dimensions. This is true regardless of the increased accuracy of 2CM or MF relative to traditional 1CM. Thus, a limitation of our study is that, while it improves the accuracy of basal diameter calculations, it does not eliminate every potential problem related to incorporating ultrasound into the measurement of basal diameter.

Another approach that can be used to account for flat areas of pigment is transillumination of the globe. Pigmented portions of the tumor can often be seen by transillumination, even when they are “flat.” However, there are certain reasons that this approach was not incorporated into our study. Because the goal of this paper was to assess the 2CM and MF methods, we wanted to use approaches that would be applicable for both posterior choroidal tumors (using B scan) as well as anterior ciliary body tumors (using UBM). Transillumination can only be utilized to determine tumor basal diameter preoperatively for those tumors located anterior enough that the entire shadow can be appreciated in clinic in an awake patient. In addition, amelanotic tumors, especially portions that are thin or flat, do not transilluminate well.

Unfortunately, recurrence due to inadequate initial plaque coverage does not appear to be a very rare occurrence. One of the authors (ABD) has been referred several patients with recurrences following brachytherapy. In all cases, the original ultrasounds and tumor LBD measurements were reviewed; and in all cases, use of the 1CM method by the referring doctor led to selection of an undersized plaque (Fig. 4).

## Conclusions

Determining LBD by a single-chord length from a B scan ultrasound is an inaccurate means of determining actual LBD, especially for larger tumors. Using either our 2CM or our MF is much more accurate, especially for tumors > 12 mm, where a single-chord ultrasound method is more likely to lead to incorrect, undersized plaque selection. Our MF can be applied with great accuracy even in cases where the eye’s AL is not measured. However, there is still the need to incorporate ophthalmoscopically-evident (but ultrasonographically-invisible) areas of flat pigment into plaque selection decisions, and so we recommend that these tumors be managed by clinicians with expertise and experience in this area.
